# Septic Pelvic Thrombophlebitis Mimicking Acute Appendicitis: A Diagnostic Challenge and the Role of Therapeutic Anticoagulation

**DOI:** 10.7759/cureus.85662

**Published:** 2025-06-09

**Authors:** Emmanuel Afful, Tajudeen Dabiri, Gary Markoff, Carolina Martinez, Dara Forrester

**Affiliations:** 1 Obstetrics and Gynecology, Bronx-Lebanon Hospital/Icahn School of Medicine at Mount Sinai, Bronx, USA

**Keywords:** c-reactive protein trending, heparin therapy, postpartum fever differential diagnosis, postpartum sepsis, septic thrombophlebitis

## Abstract

Septic pelvic thrombophlebitis (SPT) is a rare postpartum complication, occurring in approximately one in 9,000 vaginal deliveries. Known risk factors include hypertensive disorders of pregnancy, multiple gestation, nulliparity, maternal age under 20, Black race, cesarean delivery, and chorioamnionitis. Due to the lack of standard diagnostic criteria, SPT can easily be missed. We present the case of a 34-year-old woman who underwent an uncomplicated vaginal birth after cesarean (VBAC). On postpartum day 4, she developed clinical signs of endometritis, with a normal white blood cell count but elevated neutrophil percentage. Her symptoms resembled acute appendicitis on imaging, although her Alvarado score was 4. Initial treatment with empiric antibiotics and prophylactic enoxaparin was ineffective. However, clinical improvement was seen after initiating therapeutic enoxaparin. Treatment response was monitored using objective markers such as C-reactive protein (CRP) trends, neutrophil percentage, fever resolution, and subjective improvement in abdominal tenderness. This case highlights endometritis as a risk factor for SPT and underscores the importance of early therapeutic anticoagulation when SPT is suspected.

## Introduction

Septic pelvic thrombophlebitis (SPT) is a rare complication of cesarean delivery. It is rarer still among women with spontaneous vaginal delivery, 1:800 and 1:9000, respectively [1]. This may occur in the absence of known risk factors such as hypertensive disorders of pregnancy, multiple gestation, nulliparity, age less than 20 years, Black race, cesarean delivery, induced abortion, chorioamnionitis, and endometriosis [1], further complicating the diagnosis. Traditionally, the diagnosis of SPT is considered in the setting of endometritis that remains unresponsive to appropriate antibiotic therapy. The presumptive diagnosis may be confirmed by a response to antibiotic and anticoagulation therapy within 12-72 hours [2]. However, other studies have challenged this time-honored rule [3]. Proposed criteria for the diagnosis of SPT include a persistent enigmatic febrile course (>72 hours) despite broad-spectrum antibiotic therapy (>48 hours), and no evidence of an infectious etiology on culture or radiologic study [3].

Septic thrombophlebitis has been postulated as a continuum of postpartum pelvic infections, such as endometritis. A temporal relationship has been established between the onset of SPT, relative to the diagnosis of endometritis, and the identification of infectious agents in the endometrium, accompanied by signs of infection or inflammation in the affected vasculature. These findings support the endometritis-SPT hypothesis [4,5,6]. There were two times increased odds of SPT among patients with endometritis, especially following cesarean section [7], although there is also the possibility of an initially existing SPT misdiagnosed as endometritis [5].

Historically, septic thrombophlebitis was diagnosed through operative intervention. Advances in radiologic technologies have enabled SPT to be diagnosed before surgical exploration [1]. Despite these radiologic improvements, significant morbidities are still associated with SPT, such as deep vein thrombosis and acute pulmonary embolism [1]. The right-sided preponderance, lower abdominal, and right-lower-quadrant tenderness associated with SPT make acute appendicitis an appropriate differential diagnosis [5].

We present a case of postpartum septic thrombophlebitis following a spontaneous vaginal delivery that mimicked acute appendicitis.

## Case presentation

A 34-year-old woman with a history of vaginal birth after cesarean section at 39 6/7 presented to the labor and delivery (L&D) triage with a report of uterine contraction and spontaneous rupture of membranes 50 minutes before presentation. Her prenatal course was significant for chronic inactive hepatitis B and a successful vaginal birth after cesarean section, negative for Group B streptococcus.

At presentation, her vital signs were stable, and she was fully dilated, with +2 station, clear non-malodorous amniotic fluid. A male infant with Apgar scores of 9/10 and 9/10, weighing 3300 g, was delivered after nine minutes of presentation, followed by an unremarkable placental delivery. She sustained a first-degree laceration, which was repaired under local anesthesia.

She was admitted to the postpartum floor with an unremarkable early postpartum course.

On day 1 postpartum, she had no complaints and experienced normal lochia color and quantity, with uterine size at the level of the umbilicus. The patient reported mild abdominal pain on the second day postpartum, but with adequate pain control. The examination revealed some uterine tenderness. She was discharged home on postpartum day 2 pending postpartum follow-up and Gastroenterology follow-up due to chronic hepatitis B.

The patient presented to the L&D triage two days after discharge (postpartum day 4) with marked lower abdominal pain without fever (temperature of 98.6 °F) or urinary tract symptoms and normal lochia. There was marked uterine tenderness with purulent cervical discharge. There was no demonstrable adnexal mass. A bedside sonogram demonstrated a thin endometrial stripe. Empiric treatment for postpartum endomyometritis was started with intravenous clindamycin, gentamicin, 0.9% saline, and ketorolac.

**Figure 1:**
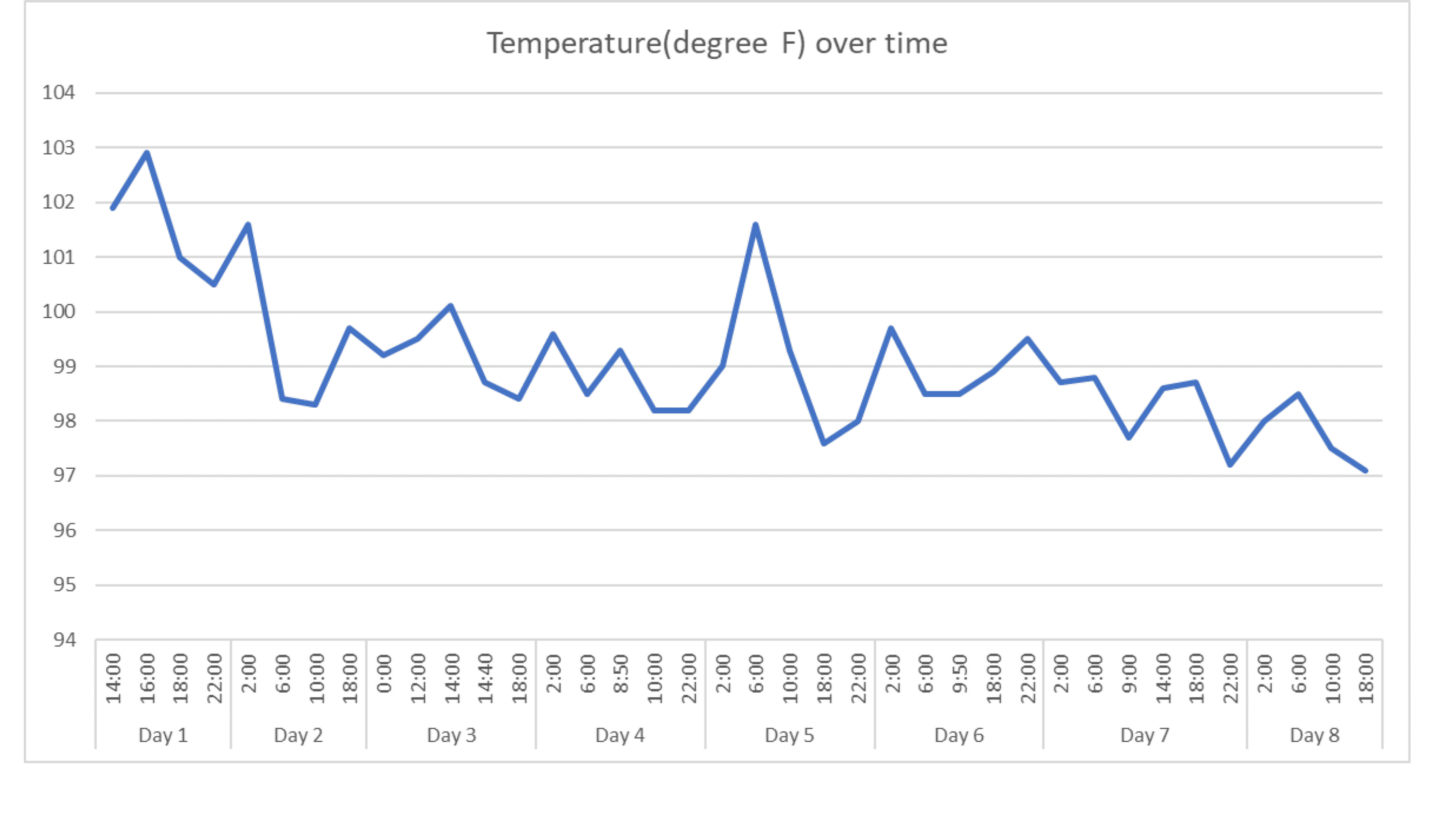
Temperature trend from day of admission and time. Therapeutic dose of enoxaparin commenced at 22:00 hour on day 4.

Two hours into admission, she had a temperature of 102.9°F, a pulse rate of 128 bpm, and a blood pressure of 84/51 mmHg. Intravenous fluid resuscitation continued while the ICU and the Infectious disease teams were consulted. Their recommendations included a CT scan of the abdomen and pelvis, cessation of clindamycin and gentamycin, and a switch to vancomycin, piperacillin/tazobactam, and doxycycline. She was admitted to the ICU due to sepsis. See Figure 1 for the temperature trend.

**Figure 2:**
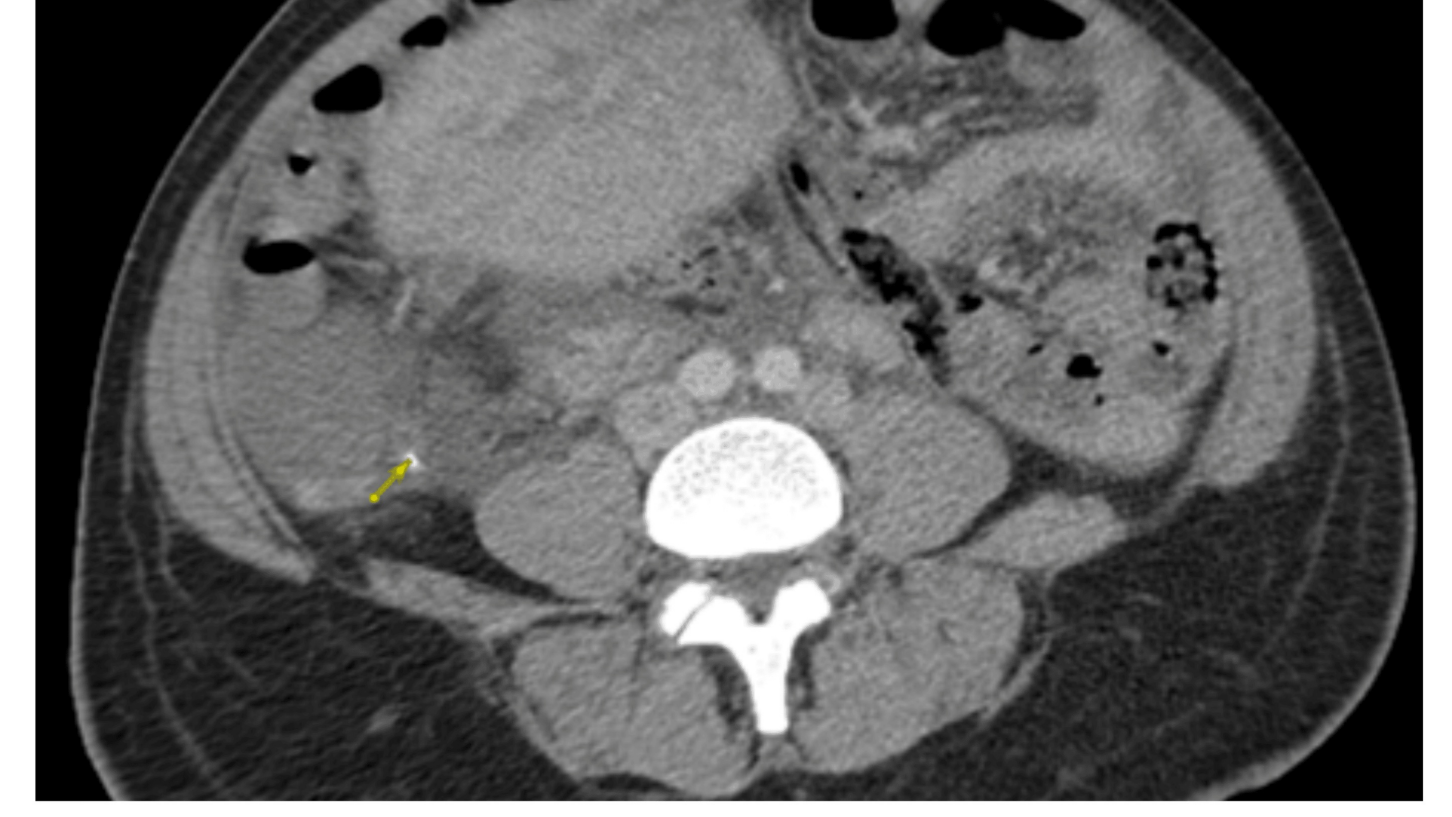
Computed tomography (CT) scan with intravenous contrast showing a dilated appendix of diameter of 1.3 cm. Seen is an appendicolith at the tip of the appendix and mild peri-appendiceal inflammation.

On the second day of readmission, a CT abdomen and pelvis with and without contrast was suspicious for acute appendicitis (see Figure 2).

**Figure 3:**
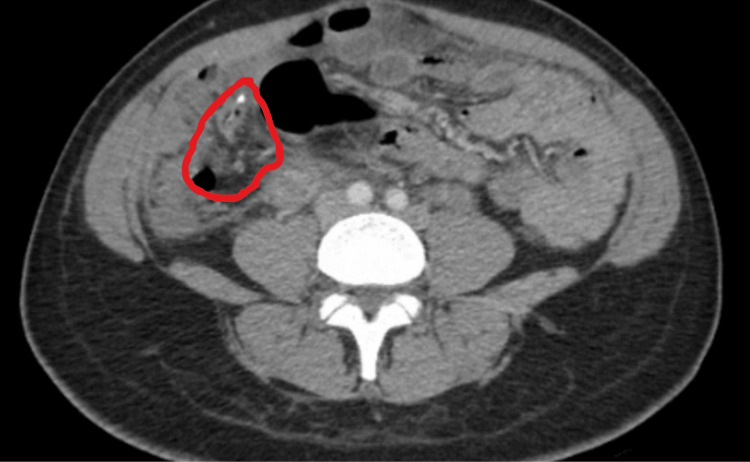
Follow-up CT abdomen and pelvis with contrast. Dispersed calcifications, ill-defined, disrupted wall of the appendix.

General surgery was consulted, and non-surgical management was recommended. The temperature remained labile (Tmax 102.9 F) despite antibiotics and prophylactic anticoagulation (enoxaparin 40 mg daily). A follow-up CT abdomen and pelvis reported that the previously identified appendicolith and calcifications appeared dispersed, and the appendiceal wall was ill-defined and disrupted (see Figure 3).

On the fourth day of admission, therapeutic anticoagulation with enoxaparin 80 mg every 12 hours was initiated, and the patient's temperature subsequently stabilized (Figure 1). She continued to receive a therapeutic dose of enoxaparin, gentamicin, and zosyn for 48 hours after the temperature settled. Inflammatory markers, particularly C-reactive protein (CRP) and the percentage of neutrophils, showed a downward trend. 

**Figure 4:**
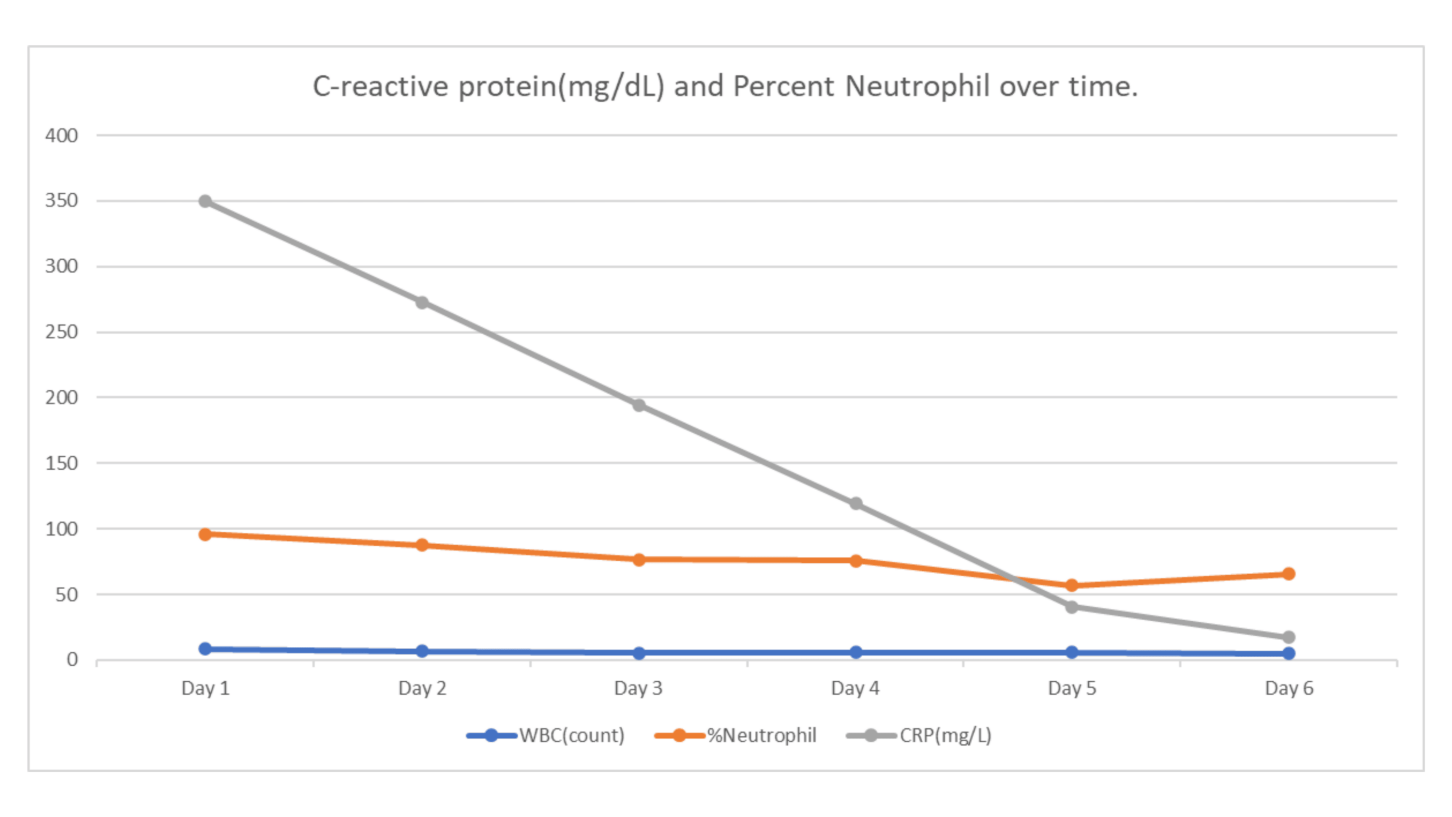
Consecutive decrease in C-reactive protein and percent neutrophil count. The total WBC count witnessed no significant changes.

She was discharged home on oral cefuroxime 500 mg twice daily for seven days and metronidazole 500 mg twice daily for seven days due to the suspicion of appendicitis. See Figure 4 for the trend of inflammatory markers.

## Discussion

SPT is a rare postpartum complication, more associated with cesarean delivery relative to vaginal delivery. The most identified risk factors for this complication have been identified by case reports and case series. One large case-control study identified additional risk factors such as age <20 years, non-Hispanic Black race, hypertensive disorders in pregnancy, public insurance, multiple gestation, chorioamnionitis, labor, and cesarean section. The highest odds of SPT were observed with cesarean section (aOR = 6.25; CI = 1.94-20.1) [1].

There is an overlap in the symptomatology of SPT and ovarian vein thrombosis (OVT). Abdominal pain has been proposed to be more severe in OVT compared to SPT [8]. However, the subjectivity of pain makes it an unreliable symptom for differentiating between conditions. In addition, signs of sepsis may not be entirely associated with OVT, as suggested by Munsick [9]. The presence of a palpable rope-like abdominal mass, as reported in most cases by Brown [5], may represent an advanced OVT. Therefore, radiologic evidence remains the ideal diagnostic tool for differentiating between SPT and OVT. In our case, no radiological findings suggest septic venous thrombosis, either at the initial CTAP or the follow-up imaging.

A CT abdomen and pelvis with intravenous contrast reported an enlarged uterus in keeping with the postpartum state and a dilated, fluid-filled appendix measuring 1.3 cm with mild hyper-enhancement and appendicolith but without significant peri-appendiceal inflammation. A follow-up CTAP four days later reported dispersed calcifications in the wall of the appendix, which was concerning for ruptured appendicitis. The ovarian veins were not optimally evaluated due to the timing of the scan.

The CTAP report heightened the suspicion of ruptured appendicitis in the setting of lower abdominal tenderness and intermittent fever. General surgery continued to follow the patient, and at one point, surgical management was contemplated but resorted to non-surgical management due to Alvarado's score of 4 and clinical picture not commensurate with ruptured appendicitis.

The initiation of a therapeutic dose of enoxaparin and the subsequent settling of the temperature were key to avoiding surgical management of the suspected ruptured appendicitis. Anticoagulation was continued for 48 hours after the temperature had stabilized. Therapeutic anticoagulation, in addition to antibiotic therapy, has been the mainstay treatment for SPT [5,8,10]. Systemic anticoagulation with either unfractionated heparin with a loading dose of 5000 IU, followed by a continuous infusion of 16-18 IU/Kg to a PTT target of 1.5-2.0 times the patient’s baseline, or low-molecular-weight heparin (1 mg/kg every 12 hours) is the recommended treatment [3,10]. The duration of anticoagulation is dependent on radiographic evidence of thrombosis. Anticoagulation may continue for two to six weeks if there is evidence of pelvic vein thrombosis, and a much longer duration may be considered for those with thrombosis outside the pelvis [10].

Antibiotic therapy should be active against streptococci, Enterobacteriaceae, and anaerobic bacteria [10]. Zosyn and gentamicin were used in this case, covering the most common organisms. There was, however, no defervescence until therapeutic anticoagulation was added. The duration of antibiotics is guided by the resolution of symptoms and clinical improvement, including defervescence and resolution of leukocytosis. The total white blood cell count was not elevated in this case. However, the percent neutrophil count and the C-reactive protein decreased proportionally to the patient’s clinical response (Figure 3). Severely elevated CRP levels (>50 mg/dL) are associated with bacterial infections in approximately 90% of cases [11]. Along with elevated body temperature, CRP can serve as a marker of infection [12]. We recommend using CRP, the percentage of neutrophils, defervescence, and other clinical parameters to monitor the response to treatment in SPT.

## Conclusions

SPT must remain high on the differential in postpartum patients with persistent fever and abdominal pain, even in the absence of classic risk factors or imaging evidence. In this case, the timely initiation of therapeutic anticoagulation led to a rapid clinical resolution, thereby sparing the patient from unnecessary surgical intervention. Serial trends in C-reactive protein and percent neutrophil count closely paralleled clinical improvement, providing objective support for management decisions. Our findings emphasize the importance of a combined clinical and biomarker-driven approach for the prompt recognition and successful treatment of SPT.
